# Supplementary material for: Hsp90‐mediated regulation of DYRK3 couples stress granule disassembly and growth via mTORC1 signaling
Source: EMBO Rep. 2021 Mar 19;22(5):e51740. doi: 10.15252/embr.202051740 (PMC8097338; doi:10.15252/embr.202051740)
Supplement: Supplementary file 1 — Expanded View Figures PDF [file EMBR-22-e51740-s002.pdf]

Expanded View Figures

Figure EV1. Inhibition of Hsp90 delays SG dissolution independently of Hsp70. Related to Fig 1.

A Kinetics of SG dissolution of living cells in absence and presence of ammonium chloride. G3BP2-GFP HeLa-Kyoto cells were treated with sodium arsenite (50 μM) for 45 min. Then, cells were allowed to recover in drug-free medium (recovery control) or in presence of ammonium chloride (NH_4Cl, 20 mM). Images were taken over a time period of 4 h every 10 min. Dashed lines = 95% confidence intervals. Number of cells counted: 193 (Recovery Control); 104 (NH_4Cl, 20 mM).

B G3BP2-GFP HeLa-Kyoto cells were either left untreated (control) or treated with GA (5 μM), 17AAG (5 μM) or VER (40 μM) for 4 h. Cells were fixed, and the percentage of cells with SGs was counted. n = 3 independent experiments, ± s.e.m.; 126–185 cells counted/sample. n.s.: non-significant (one-way ANOVA).

C, D Kinetics of SG dissolution of living HEK293 cells that express the SG-resident protein PABP endogenously tagged with the fluorescent probe Dendra2 (PABPC1-Dendra2). PABPC1-Dendra2 HEK293K cells were treated with sodium arsenite (100 μM) for 45 min. Then, cells were allowed to recover in drug-free medium (recovery control) or in presence of ammonium chloride (NH_4Cl, 20 mM), GA (5 μM), 17AAG (5 μM), or VER (40 μM). Images were taken over a time period of 4 h every 10 min. Dashed lines = 95% confidence intervals. Number of cells counted: 299 (recovery control); 160 (NH_4Cl, 20 mM); 361 (GA 5 μM); 193 (17AAG 5 μM); and 259 (VER 40 μM).

E Confocal microscopy showing that Hsp90 does not colocalize with DRiPs. HeLa cells were treated with OP-puro (25 μM) for 1 h. DRiPs were visualized by click chemistry, while Hsc70, Hsp70, Hsp90 α, and β were visualized by immunostaining. Scale bar is 10 μm.

F Quantitation of DRiP enrichment in SGs. Automated imaging and SG segmentation are based on G3BP signal. Data are presented as histogram. HeLa cells were treated with HS at 43.5°C for 1 h alone or in presence of GA (5 μM), 17AAG (5 μM), or VER (40 μM). Number of SGs segmented: 2,198 (control); 3,056 (VER); 1,484 (GA); 3,220 (17AAG); $P < 10^{-10}$ (one-way ANOVA).

G, H HeLa cells stably expressing V5-tagged inducible Hsp70 under the control of tetracycline (Flp-In) were cultured in absence or presence of tetracycline (V5-HSP70 OFF and V5-HSP70 ON, respectively). (G) Protein extracts were prepared 24 h after treatment, and Hsp70 levels were analyzed by immunoblotting. TUBA4A was used as loading control. (H) Cells were treated with sodium arsenite (500 μM) for 45 min, followed by recovery in drug-free medium (control) or in presence of VER (40 μM), GA (5 μM) or 17AAG (5 μM) for 90 min. Cells were then fixed, and the percentage of cells with persisting SGs was counted. n = 3 independent experiments, ± s.e.m.; 115-378 cells counted/sample; P = n.s.: non-significant (one-way ANOVA).

I HeLa cells were either left untreated or treated with VER (40 μM), GA (5 μM), or 17AAG (5 μM) for 4 h or 24 h, respectively. Cells were fixed and stained for endogenous DCP1A, a marker of P-bodies. Nucleic acid was stained with DAPI. Quantitation of the percentage of cells with P-bodies after 4 or 24 h of treatment is shown. n = 3 independent experiments, ± s.e.m.; 4 h: 104–124 cells counted/sample, P = 0.02; 24 h: 111–178 cells counted/sample, $P < 10^{-7}$ (one-way ANOVA).

J G3BP2-GFP HeLa-Kyoto cells were lipofected with a cDNA encoding for mRFP-DCP1A. 24 h post-transfection, cells were treated with sodium arsenite (50 μM) for 45 min. Then, cells were allowed to recover in drug-free medium (recovery control) or in presence of GA (5 μM), 17AAG (5 μM), or VER (40 μM). Images were taken over a time period of 2 h every 10 min. Representative images of GFP-G3BP2 SGs and mRFP-DCP1A P-bodies at 0, 30, 60, 90, and 120 min of recovery time are shown. Scale bar is 10 μm.

© 2021 The Authors

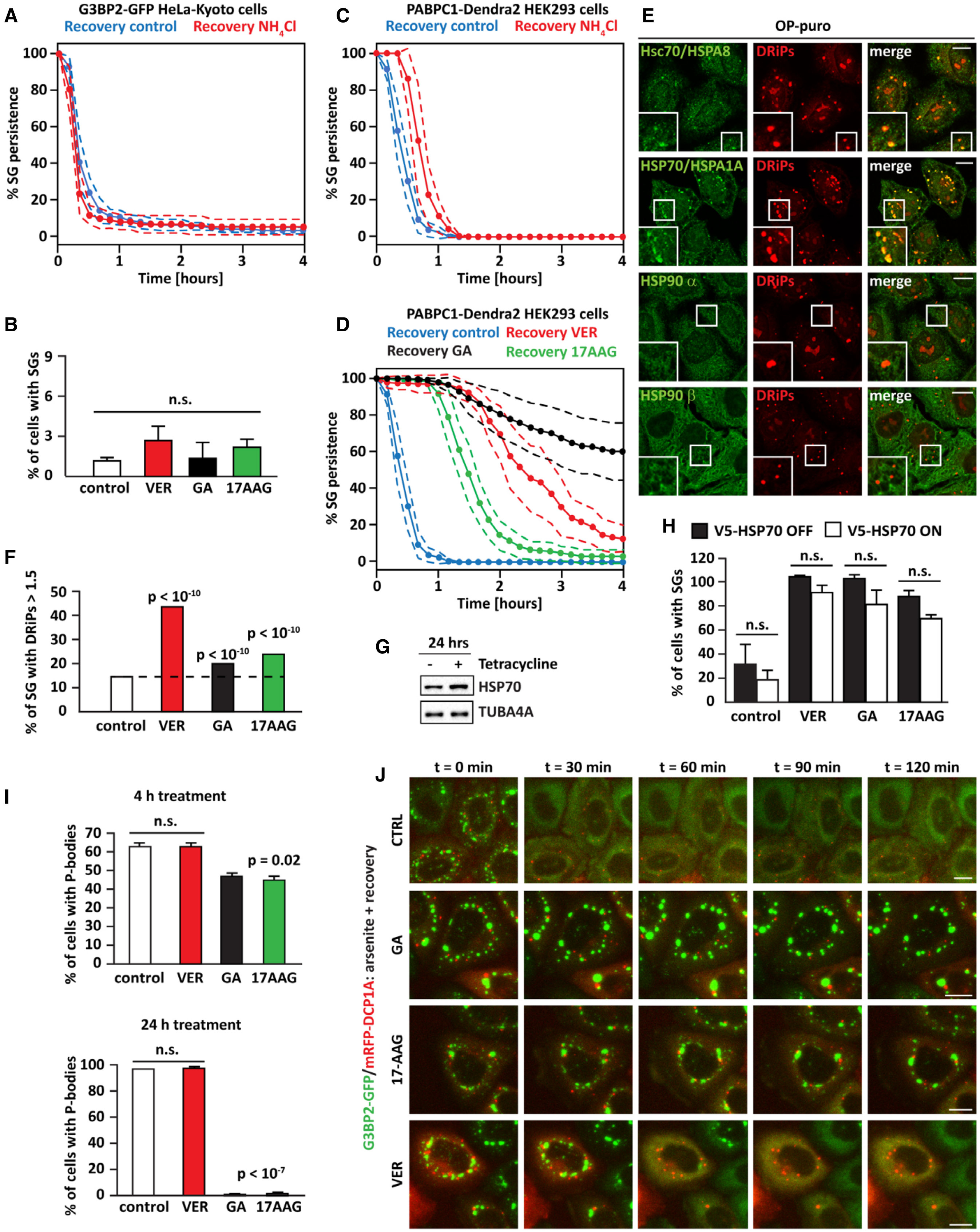

Figure EV1.

 © 2021 The Authors

Figure EV2. Raptor is a client of Hsp90. Related to Fig 2 and Movie EV6.

A HeLa cells were subjected to PLA using antibodies specific for endogenous Hsp90 or DYRK3. Negative controls (no antibodies, Hsp90 antibody, or DYRK3 antibody alone) are shown. Scale bar is 10 μm.

B HeLa cells were lipofected with a cDNA encoding for GFP-DYRK3. 24 h post-transfection, the cells were subjected to PLA using antibodies specific for endogenous Hsp90 and DYRK3. PLA foci and nuclei were segmented, and PLA foci/cell were automatically quantified. The mean of PLA/foci in cells incubated with no antibodies (−), only Hsp90 antibody, only DYRK3 antibody, and both Hsp90 and DYRK3 antibodies is shown. Cells incubated with Hsp90 and DYRK3 antibodies were used as control. $n = 3$ independent experiments, ± s.e.m.; 235–359 cells counted/sample, $P < 10^{-8}$ (one-way ANOVA). Scale bar is 10 μm.

C HeLa cells were lipofected with a cDNA encoding for myc-raptor. 6 h post-transfection, cells were incubated in drug-free medium (−) or in presence of GA, 17AAG, or VER (concentrations are shown). Total proteins were extracted 16 h later. Myc and TUBA4A protein levels were analyzed by immunoblotting. $n = 3$ independent experiments, ± s.e.m.; $P < 10^{-5}$ (one-way ANOVA).

D HeLa-Kyoto cells were transfected with vectors coding for GFP-DYRK3 and mCherry Hsp90 (α and β). 24 h post-transfection, cells were either left untreated (control) or treated with GA (5 μM) and subjected to live-cell imaging. Cells were imaged for up to 4 h, taking pictures every 10 min. Selected time points are shown. The arrowheads indicate GFP-DYRK3 condensates that are devoid of mCherry Hsp90. Control: scale bar is 5 μm; GA: scale bar is 10 μm.

E HeLa-Kyoto cells stably expressing G3BP2-GFP were left untreated (control) or treated with sodium arsenite (500 μM) for 45 min. Where indicated cells were allowed to recover in drug-free medium or in presence of GSK621666 (5 μM) for 2 or 4 h. In addition, cells were treated with GSK621666 (5 μM) for 4 h, without previous exposure to arsenite (GSK 4 h). Cells were fixed and the percentage of cells with SGs was counted. Number of cells counted: 576 (control); 595 (ars.); 427 (ars. + recovery drug-free medium 2 h); 387 (ars. + recovery with GSK 2 h); 654 (ars. + recovery drug-free medium 4 h); 610 (ars. + recovery with GSK 4 h); and 298 (GSK 4 h). $n = 3–5$ independent experiments, ± s.e.m.; $P = 10^{-3}$ (one-way ANOVA).

F, G G3BP1-mCherry HeLa-Kyoto cells were lipofected for 72 h with a non-targeting siRNA control or siRNAs specific for DYRK3. (F) Efficacy of DYRK3 knockdown was verified in total protein extracts. TUBA4A was used as loading control. (G) Cells were treated with sodium arsenite (50 μM) for 45 min, followed by recovery in presence of GA (5 μM). Kinetics of SG dissolution are reported. Images were taken over a time period of 4 h every 10 min. Dashed lines = 95% confidence intervals. Number of cells counted: 866 (siRNA control) and 854 (siRNA DYRK3).

© 2021 The Authors

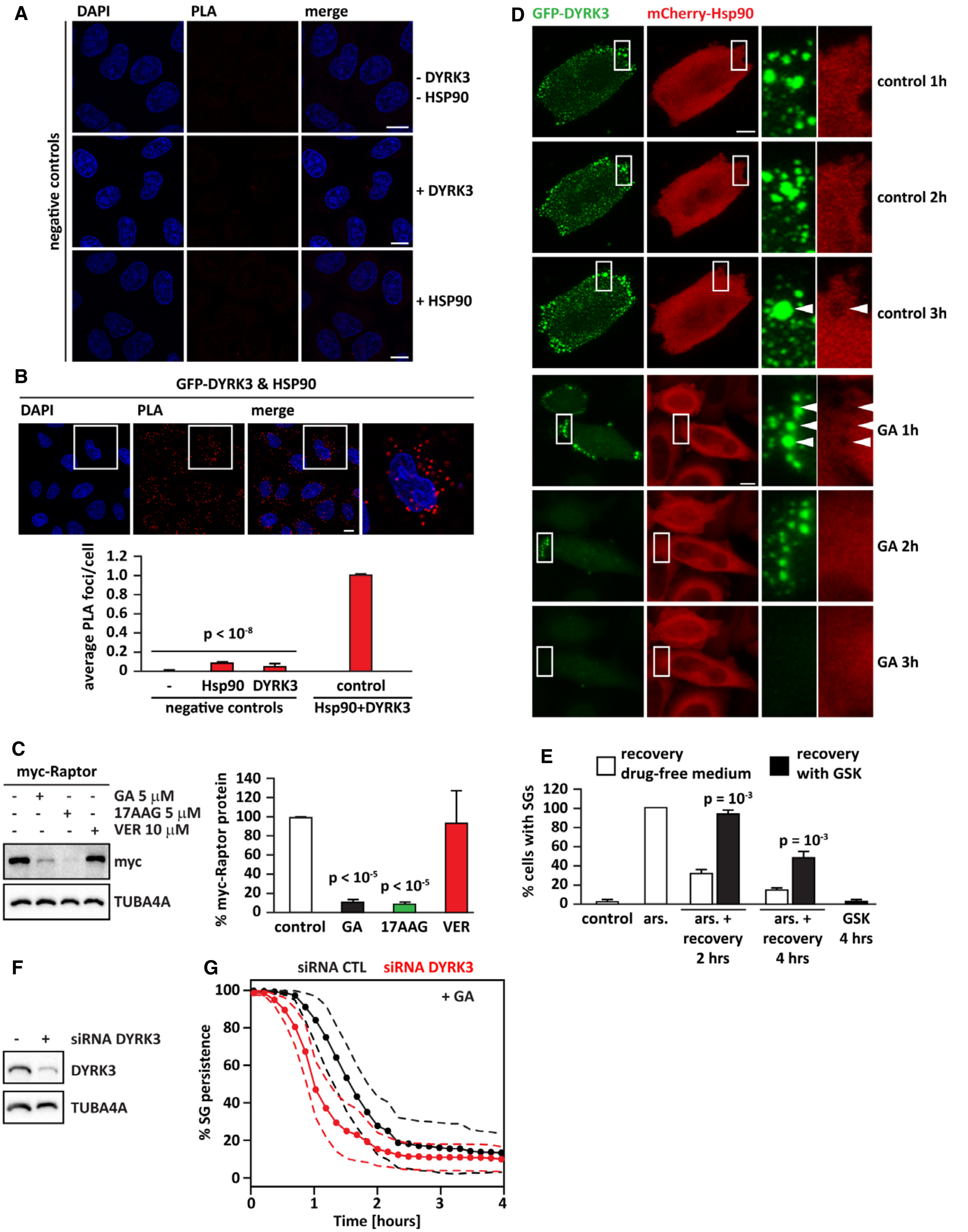

Figure EV2.

© 2021 The Authors

Figure EV3. Upon inhibition, DYRK3 relocalizes to splicing speckles and promotes aberrant SC35 mitotic body accumulation. Related to Fig 3.

A–C HeLa cells were left untreated or treated with GSK621666 (1 μM) for 2 h. Cells were fixed and stained for DYRK3 and SC35. Scale bar is 10 μm. (B) Automatic segmentation of nuclear speckles is based on SC35. An automated imaging assay was used to quantify the percentage of nuclear speckles highly enriched for DYRK3 (using a fluorescent ratio 1.5 as threshold). Number of speckles quantified = 5,600 (control); 9,794 (GSK 2 h); $P < 10^{-10}$ (Student's *t*-test). (C) Quantification of the percentage of mitotic cells showing aberrant SC35 mitotic bodies. $n = 4$ independent experiments, ± s.e.m. Number of cells counted: 719 (control); 720 (GSK 2 h); $P = 0.0001$ (Student's *t*-test).

D HeLa cells were left untreated (control) or treated with GA (5 μM), 17AAG (5 μM), or VER (10 μM) for 8 h. Cells were then fixed and immunostained for SC35 and P-Ser10-H3 antibodies. Scale bar is 10 μm.

E HeLa cells were left untreated or treated with GSK621666 (1 μM) or GA (5 μM) for 2 h. Cells were fixed and stained for DYRK3 and Hsp90. Scale bar is 10 μm.

F HeLa cells overexpressing GFP-DYRK3 were fixed 24 h post-transfection. Nucleoli were visualized with an antibody specific for fibrillarin. Scale bar is 10 μm.

G HeLa cells were lipofected with a cDNA encoding for GFP-DYRK3 or GFP-DYRK3-dN. 24 h post-transfection, cells were either left untreated or exposed to GA (5 μM) for 8 h. The subcellular distribution of GFP-DYRK3 and GFP-DYRK3-dN was investigated by confocal microscopy. Splicing speckles were visualized with an antibody specific for SC35. Scale bar is 10 μm.

H 24 h post-transfection, HeLa cells overexpressing GFP-DYRK3 or GFP-DYRK3-dN were lysed and cell lysates were subjected to immunoprecipitation of GFP proteins. Levels of GFP and endogenous Hsp90 are shown in the input and bead fractions.

I Replacing the N-terminus of DYRK3 with the Sup35-NM domain (Sup35-NM-GFP-DYRK3-dN) restores DYRK3 targeting to stress granules. 24 h post-transfection, HeLa cells overexpressing Sup35-NM-GFP-DYRK3-dN were either left untreated or exposed to arsenite (500 μM) for 45 min. Sup35-NM-GFP-DYRK3-dN recruitment inside SGs was investigated by confocal microscopy. SGs were visualized with an antibody specific for G3BP. Scale bar is 10 μm.

J Confocal microscopy image of HeLa cells expressing for 24 h Sup35-NM-GFP-DYRK3-dN and either left untreated (control) or exposed to GA (5 μM) for 8 h. DAPI was used to stain nucleic acid. Scale bar is 10 μm.

K Total proteins were extracted from HeLa cells treated as described in (J); GFP and TUBA4A protein levels were analyzed by immunoblotting.

© 2021 The Authors

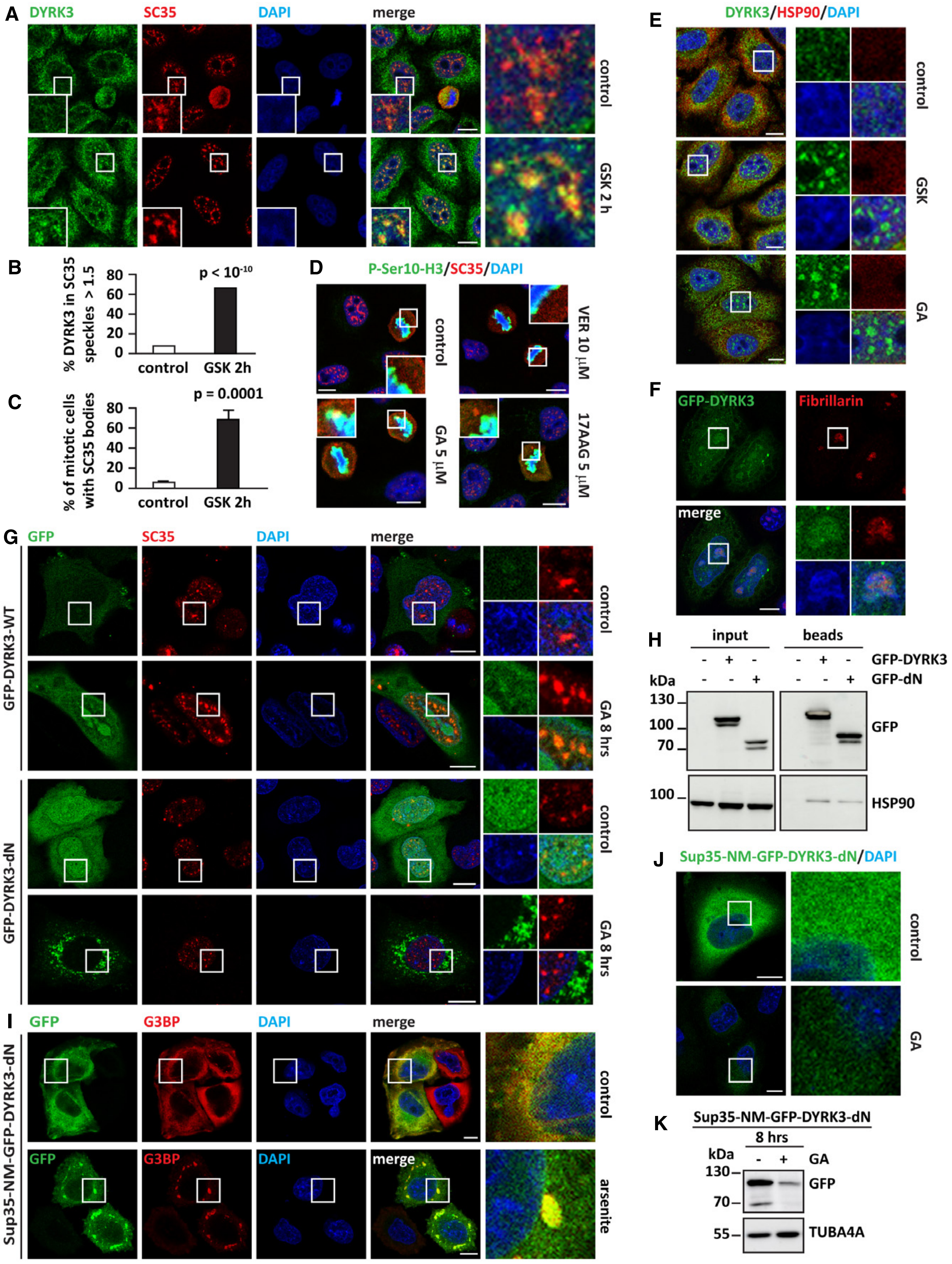

Figure EV3.

 © 2021 The Authors

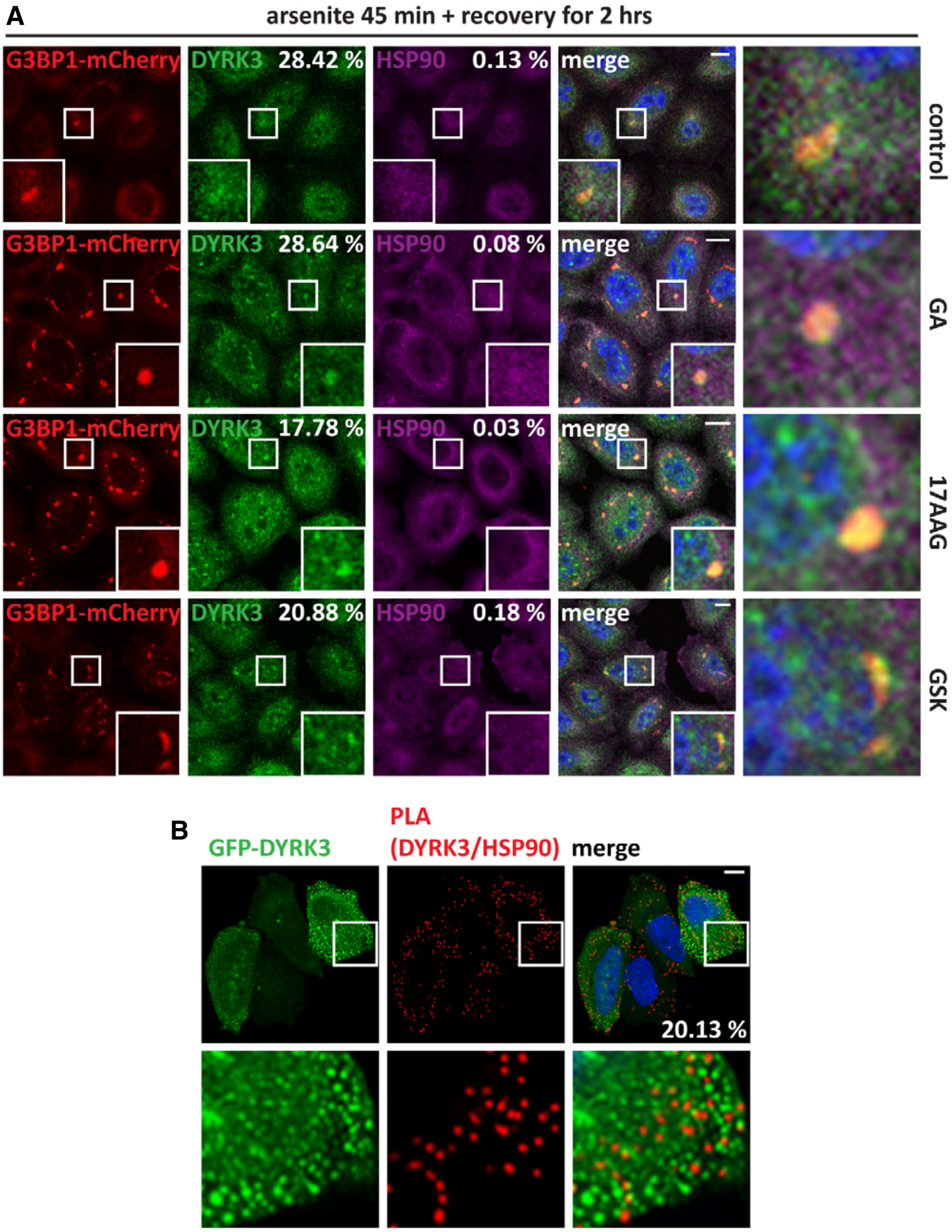

Figure EV4. Hsp90 does not colocalize with DYRK3 inside persisting SGs, nor with the pool of condensed GFP-DYRK3. Related to Fig 4.

A G3BP1-mCherry HeLa-Kyoto cells were left untreated or treated with sodium arsenite (50 μM) for 45 min to induce SGs, followed by recovery in drug-free medium or in presence of GA (5 μM), 17AAG (5 μM), or GSK (5 μM) for 2 h. Cells were then fixed and immunostained for DYRK3 and total Hsp90 antibodies. Quantitation of the percentage of SGs that shows DYRK3 and Hsp90 enrichment (> 1.5). Automated imaging and SG segmentation is based on mCherry-G3BP1 signal. Number of SGs analyzed: 774 (control), 2,817 (17AAG), 1,187 (GA), and 936 (GSK/GKS626616). Scale bar is 10 μm.

B Confocal microscopy showing the distribution of GFP-DYRK3 and PLA foci indicative of Hsp90-DYRK3 interaction. Quantitation of the percentage of PLA foci adjacent to/partly colocalizing with GFP-DYRK3 condensates is indicated. Automated imaging and segmentation of GFP-DYRK3 condensates (n = 2,126). Scale bar is 10 μm.

Figure EV5. Hsp90 couples SG dissolution and translation restoration via DYRK3.

A HeLa-Kyoto cells were left untreated or treated with sodium arsenite (50 μM; A) for 45 min to induce SGs, followed by recovery in drug-free medium or in presence of LY294002 (50 μM) for 4 h. Where indicated cells were only treated with LY294002 (50 μM) for 4 h. Expression levels of total and phosphorylated p70 S6K and PRAS40 were investigated by immunoblotting. TUBA4A was used as loading control.

B HeLa-Kyoto cells were treated as described above except that Wortmannin (200 nM) was used instead of LY294002 (50 μM).

C, D Confocal microscopy on G3BP1-mCherry HeLa-Kyoto cells overexpressing myc-Raptor were left untreated or treated with sodium arsenite (ARS; 50 μM) for 45 min to induce SGs, followed by recovery in drug-free medium or in presence of GA (5 μM), 17AAG (5 μM, high or 0.5 μM, low) for 2 h. Scale bar is 10 μm.

E HeLa-Kyoto cells were treated as described above, and protein extract was prepared at the indicated time points. Expression levels of p70 S6K, PRAS40, and 4E-BP1, total and phosphorylated forms, were investigated by immunoblotting. TUBA4A was used as loading control.

F Impact of treatments of GA (5 μM), 17AAG (5 μM, high or 0.5 μM, low) for 4 h on total and phosphorylated p70 S6K, PRAS40, and 4E-BP1.

G HeLa-Kyoto cells were treated with sodium arsenite (50 μM) for 45 min to induce SGs, followed by recovery in drug-free medium or in presence of GA (5 μM), 17AAG (5 μM, high or 0.5 μM, low) for 1 and 4 h. Where indicated, 15 min prior to protein extraction, puromycin (5 μg/ml) was added to the medium. The total levels of puromycilated proteins were investigated by immunoblotting. TUBA4A was used as loading control.

H Working model showing that Hsp90 inhibition impairs the activation of the mTORC1 pathway via inhibition of DYRK3-mediated disassembly of SGs (SG-dependent) and inhibition of Akt (SG-independent).

Data information: Related to Movies EV8 and EV9.

Source data are available online for this figure.

© 2021 The Authors

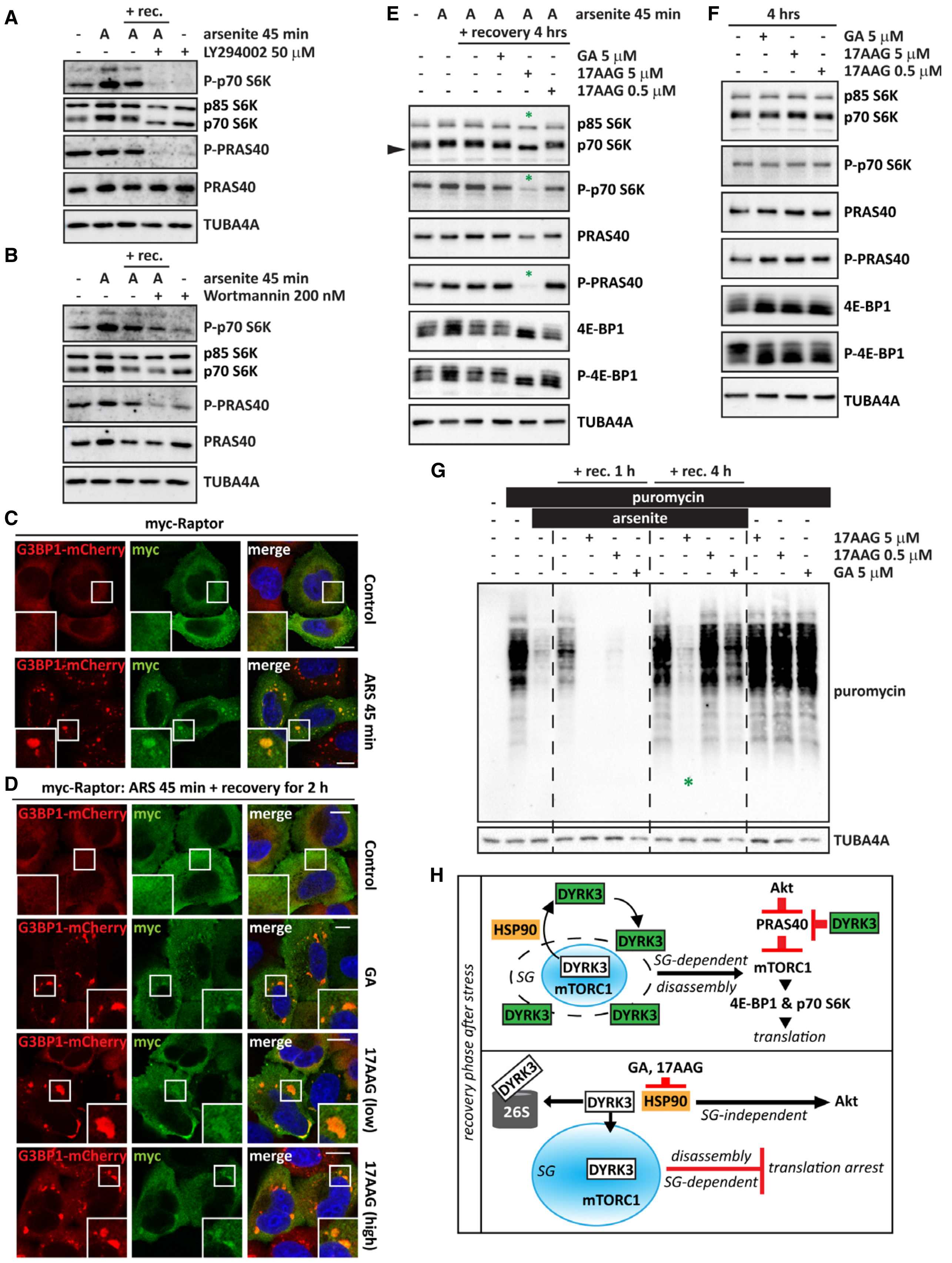
A
+ rec.
arsenite 45 min
LY294002 50 μM
P-p70 S6K
p85 S6K
p70 S6K
P-PRAS40
PRAS40
TUBA4A
B
+ rec.
arsenite 45 min
Wortmannin 200 nM
P-p70 S6K
p85 S6K
p70 S6K
P-PRAS40
PRAS40
TUBA4A
C
myc-Raptor
G3BP1-mCherry
myc
merge
Control
ARS 45 min
D
myc-Raptor: ARS 45 min + recovery for 2 h
Control
GA
17AAG (low)
17AAG (high)
E
arsenite 45 min
+ recovery 4 hrs
GA 5 μM
17AAG 5 μM
17AAG 0.5 μM
p85 S6K
p70 S6K
P-p70 S6K
PRAS40
P-PRAS40
4E-BP1
P-4E-BP1
TUBA4A
F
4 hrs
GA 5 μM
17AAG 5 μM
17AAG 0.5 μM
p85 S6K
p70 S6K
P-p70 S6K
PRAS40
P-PRAS40
4E-BP1
P-4E-BP1
TUBA4A
G
+ rec. 1 h
+ rec. 4 h
puromycin
arsenite
17AAG 5 μM
17AAG 0.5 μM
GA 5 μM
puromycin
TUBA4A
H
recovery phase after stress
DYRK3
HSP90
SG
mTORC1
SG-dependent
disassembly
Akt
PRAS40
mTORC1
4E-BP1 & p70 S6K
translation
GA, 17AAG
26S
SG-independent
disassembly
SG-dependent
translation arrest

Figure EV5.
