# Supplementary figures and images for: Hsp90‐mediated regulation of DYRK3 couples stress granule disassembly and growth via mTORC1 signaling
Source: EMBO Rep. 2021 Mar 19;22(5):e51740. doi: 10.15252/embr.202051740 (PMC8097338; doi:10.15252/embr.202051740)

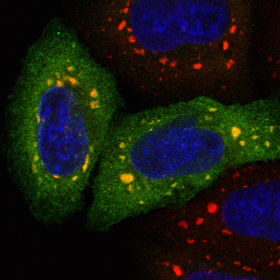

Supplement: Supplementary file 12 — Source Data for Expanded View [file EMBR-22-e51740-s005.zip › FigEV5C ARS 45 min_SEL.tif]

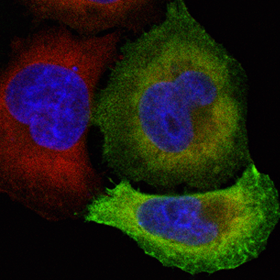

Supplement: Supplementary file 12 — Source Data for Expanded View [file EMBR-22-e51740-s005.zip › FigEV5C Control_SEL.tif]
